# The origin of human CD20^+^ T cells: a stolen identity?

**DOI:** 10.3389/fimmu.2024.1487530

**Published:** 2024-11-22

**Authors:** Marina Rode von Essen, Lisbeth Egelykke Stolpe, Helle Bach Søndergaard, Finn Sellebjerg

**Affiliations:** The Danish Multiple Sclerosis Center, Department of Neurology, Rigshospitalet, University of Copenhagen, Glostrup, Denmark

**Keywords:** CD20+ T cells, trogocytosis, endogenous CD20 production, *MS4A1*, CD20 on proliferating T cells

## Abstract

Human T cells expressing CD20 play an important role in the defense against virus and cancer and are central in the pathogenesis of both malignancies and various autoimmune disorders. Therapeutic modulation of CD20^+^ T cells and the CD20 expression level is therefore of significant interest. In rodents, CD20 on T cells is likely the product of an active transfer of CD20 from a donor B cell interacting with a recipient T cell in a process termed trogocytosis. Whether the same applies to human CD20^+^ T cells is highly debated. Investigating this dispute showed that human CD20^−^ T cells could achieve CD20 along with a series of other B-cell markers from B cells through trogocytosis. However, none of these B-cell markers were co-expressed with CD20 on human CD20^+^ T cells in blood or inflamed CSF, implying that additional mechanisms may be involved in the development of human CD20^+^ T cells. In support of this, we identified true naïve CD20^+^ T cells, measured endogenous production of CD20, and observed that CD20 could be inherited to daughter cells, contradicting that all human CD20^+^ T cells are a product of trogocytosis.

## Introduction

The peripheral T-cell pool of healthy individuals contains 3%–5% T cells expressing CD20 (1, 2). CD20^+^ T cells are of pro-inflammatory nature with a high production of IFNγ, TNFα, GM-CSF, and IL-17 (1, 3, 4). Studies indicate that CD20^+^ T cells may have a protective function in cancer (5–7) and viral infections (8–10) and a pathogenic role in CD20^+^ T-cell malignancies (11, 12) and various autoimmune disorders including multiple sclerosis (1, 13–17), psoriasis (18), and rheumatoid arthritis (19, 20). In addition to the smaller subset of T cells expressing CD20, CD20 is expressed on the surface of all B cells. Here, CD20 likely functions as part of a store-operated calcium channel to induce calcium-dependent cellular processes (21, 22), and following antigen B-cell receptor (BCR)–ligation, CD20 associates with intracellular signaling molecules to amplify signaling from the BCR (21). Whether this also applies to CD20 on T cells and T-cell antigen-receptor (TCR) signaling is unknown, but considering the increased reactivity of CD20^+^ T cells to antigen (1), it is a likely scenario.

CD20 is an important therapeutic target for the treatment of B-cell malignancies and autoimmune diseases in which B cells play a central role in disease pathogenesis. For this, therapies using monoclonal antibodies against CD20 have proven highly effective in promoting B-cell depletion and clinical improvement (23–25). In addition to B-cell depletion, anti-CD20 antibody therapy also depletes T cells expressing CD20 (26). The use of anti-CD20 antibodies to target CD20^+^ T cells in autoimmunity and CD20^+^ T-cell malignancies may therefore likewise be of great value to patients. A study investigating the efficacy of anti-CD20 antibody therapy to target CD20^+^ T-cell lymphoma showed a close relationship between killing efficiency and the surface level of CD20 (11); a challenge also acknowledged in the treatment of CD20^+^ B-cell lymphomas (27). To circumvent this, combined therapy to increase CD20 expression on B-cell lymphomas is now investigated as a method to increase the efficacy of the anti-CD20 monoclonal antibodies (28). Where CD20 expression on B cells may successfully be increased using this approach, CD20 on T cells may not, as the biology of CD20 in T cells could differ from B cells.

A recent study documented that CD20^+^ T cells in rodents did not have endogenous production of CD20 and that CD20^+^ T cells arose upon T- and B-cell interaction in which T cells acquired CD20 from B cells through trogocytosis (17). Trogocytosis is a process where two cells interact and exchange surface plasma membrane fragments, potentially transferring functional properties from one cell to another (29, 30). In the case of T- and B-cell trogocytosis, a T cell interacts with a B cell through TCR:MHC binding, actively inducing the transfer of various surface proteins from the B cell to the T cell (30–32). If CD20^+^ T cells in humans, like in rodents, exclusively are a result of trogocytosis, it would impact a possible therapeutic approach where CD20 expression or a certain level of CD20 is a prerequisite for successful therapy.

In contrast to mouse studies, human and non-human primate studies have indicated that CD20^+^ T cells may not only be a result of trogocytosis. Considering the clinical importance of this controversy, the purpose of this study was to clarify the origin of CD20^+^ T cells in humans.

## Materials and methods

### Blood and CSF samples

Venous blood from healthy individuals was collected, and peripheral blood mononuclear cells (PBMCs) were isolated by density gradient centrifugation using Lymphoprep (Axis-Shield, Oslo, Norway) and washed twice in cold phosphate buffered saline (PBS)/2 mM ethylenediaminetetraacetic acid. For cryopreservation, PBMCs were frozen in fetal bovine serum (Thermo Fisher Scientific, MA, USA)/10% dimethyl sulfoxide (DMSO; Sigma-Aldrich, MO, USA) at −150°C; some were stained with carboxyfluorescein succinimidyl ester (CFSE; Molecular Probes, MA, USA) prior to freezing. As described previously, cord blood was collected from newborns and PBMC separated as for venous blood (1). Cerebrospinal fluid (CSF) from treatment-naïve patients with relapsing remitting multiple sclerosis (RRMS) was collected in a polypropylene tube on ice and immediately centrifuged for 10 min at 400 g to separate cells from fluid. All patients were diagnosed with RRMS based on the 2017 revised McDonald criteria (33).

### Flow cytometry

Cells applied to flow cytometry were incubated with FcR blocking reagent (Miltenyi Biotec, Bergisch Gladbach, Germany) to prevent nonspecific Ab binding, and thereafter stained in PBS/2% FBS/0.02% NaN_3_ with a combination of fluorochrome-conjugated antibodies and live/dead stain (Thermo Fisher Scientific, MA, USA). Isotype-matched controls were used to correct for nonspecific Ab binding and spectral overlap, where appropriate. Data were acquired on a FACSymphony or FACSCanto II flow cytometer, both from BD Biosciences (CA, USA), and data analysis was performed using the software FlowJo (TreeStar, OR, USA). Antibodies used are listed in **Supplementary Table S1**. Gating strategy to identify CD20^+^ T cells include gating of lymphocytes in a FSC/SSC dot plot, single cells in a FSC-A/FSC-H dot plot, live cells (corresponding to cells negative for the live/dead stain), and hereafter CD20^+^ T cells in a CD3/CD20 dot plot.

### Trogocytosis

**Figure 1:**
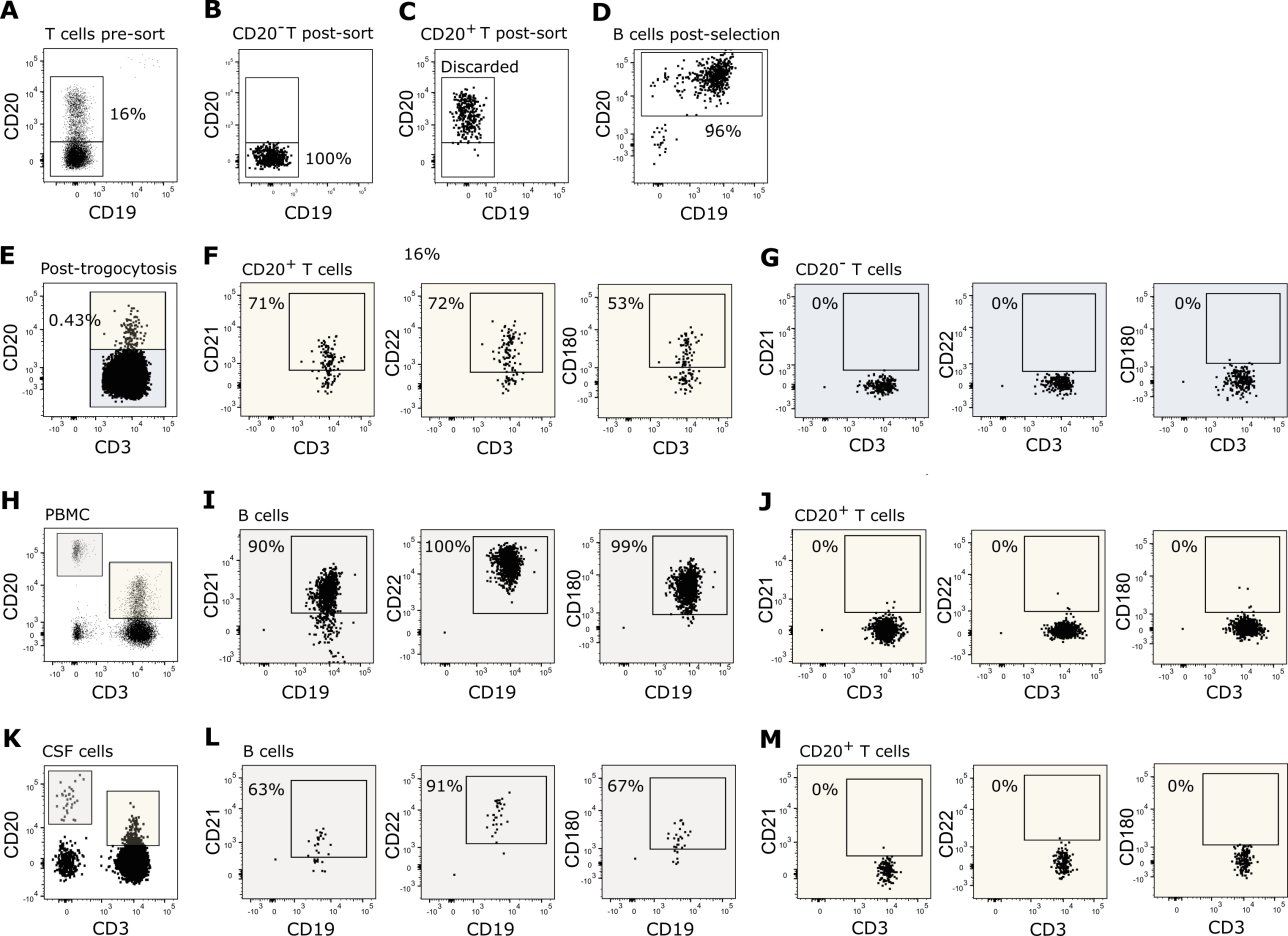
T cell trogocytosis. **(A–D)** Flow cytometry dot plot example of purified T and B cells used in the trogocytosis assay. **(A)** Negatively isolated T cells prior to fluorescence activated cell sorting of **(B)** CD20^−^ T cells and **(C)** CD20^+^ T cells. **(D)** Negatively isolated B cells. **(E–G)** Flow cytometry dot plot example of T cells after 1 h of trogocytosis. **(E)** CD20 transferred to CD20^−^ T cells following trogocytosis. Expression of CD21, CD22 and CD180 on **(F)** CD20^+^ T cells and **(G)** CD20^−^ T cells following trogocytosis. **(H–J)** Expression of CD21, CD22, and CD180 on **(I)** B cells and **(J)** CD20^+^ T cells in the blood. **(K–M)** Expression of CD21, CD22, and CD180 on **(L)** B cells and **(M)** CD20^+^ T cells in the CSF.

For trogocytosis, purified untouched B cells and untouched CD20^−^ T cells from freshly drawn blood were used. B cells were first isolated from PBMCs using a negative selection kit, followed by depletion of potential remaining T cells to ensure removal of all CD20^+^ T cells; both kits were from Stem Cell Technologies (Vancouver, Canada). The purity test showed <0.15% T cells, <0.25% NK cells, and <0.25% monocytes. For CD20^−^ T cell isolation, T cells were first negatively selected using a kit from Stem Cell Technologies followed by fluorescence-activated cell sorting based on antibodies recognizing CD20 (PE-Cy7, 2H7) to discriminate CD20^−^ from CD20^+^ T cells and CD19 (APC, HIP19) to ensure exclusion of potential remaining B cells, both antibodies from BioLegend (CA, USA). The purity of isolated CD20^−^ T cells was 100%. An example is shown in **Figures 1A–D**. To accommodate T:B cell interaction and trogocytosis, 300,000 B cells were pulsed for 20 h with 30 µg/ml glatiramer acetate and 300,000 CD20^−^ T cells added. The T:B cell co-culture was incubated for 1 h and cells analyzed by flow cytometry for identification of transferred proteins, as described above. Growth media used was RPMI 1640 (Thermo Fisher Scientific, MA, USA)/5% human AB serum (Invitrogen, MA, USA)/pen-strep (Thermo Fisher Scientific, MA, USA). The assay was performed two independent times with different donors.

### *MS4A1*-mRNA

T cells were negatively selected from three healthy donors using a kit from Stem Cell Technologies, and CD20^+^ and CD20^−^ T cells were isolated by fluorescence-activated cell sorting using antibodies targeting TCRαβ (APC; IP26) and CD20 (PE-Cy7, 2H7) both from BioLegend. The purified T cells were lysed in QIAzol (QIAGEN, Hilden, Germany) lysis buffer, chloroform added, and samples separated into an aqueous and an organic phase by centrifugation. RNA was precipitated from the aquas phase using an RNeasy kit from Qiagen and used for quantitative polymerase chain reaction (qPCR) or affymetrix analysis. For real-time qPCR analysis, synthesis of cDNA was performed on total RNA using the High-Capacity cDNA Reverse Transcription Kit (Life Technologies, Carlsbad, USA) as prescribed by the manufacturer, followed by amplification on a ViiA™ 7 real-time PCR System using TaqMan™ Fast Advanced Master Mix and commercially manufactured primer/probe kits (*MS4A1*: Hs00544819_m1, *SDHA*: Hs00417200_m1, *RNA18S5*, Hs03928989_g1) (Thermo Fisher Scientific, MA, USA). *MS4A1* and *SDHA* were amplified on undiluted cDNA, whereas a 1:1000 dilution was used for *RNA18S5*. Index qPCR values were calculated using the 2^-ΔΔ^ *^C^* ^T^ method with *SDHA* and *RNA18S5* as reference genes relative to mean *MS4A1* from CD20^−^ T cells. Affymetrix analysis was performed at the Core facility for Genomic Medicine, the Kennedy Center, Rigshospitalet, Denmark, according to the manufacturer. The array used was the Human Gene 2.0 ST Array.

### FoxO1-inhibition

Cryopreserved PBMCs from healthy controls were thawed and T cells negatively selected using a kit from Stem Cell Technologies, according to the manufacturer. Purified T cells were then cultured at a concentration of 1 × 10^6^ cells/ml for 48 h in RPMI 1640/5% human AB serum/pen-strep with 0–20 µM FoxO1-inhibitor (AS1842856; Merck, Darmstadt, Germany). Hereafter, CD20 expression on T cells was analyzed by flow cytometry as described above. The experiment was performed three independent times with a total of six individuals.

### Anti-CD3/CD28 antibody stimulation of T cells

Cryopreserved carboxyfluorescein succinimidyl ester (CFSE)–labeled PBMC from three healthy donors were thawed, with T cells negatively selected, followed by depletion of potential remaining B cells using kits from Stem Cell Technologies, according to the manufacturer. Complete removal of B cells was ensured by flow cytometry prior to T-cell cultivation in flat-bottomed plates coated over night at 4°C with 0–4 µg/ml anti-CD3 antibodies (OKT3; BioLegend) and 1 µg/ml anti-CD28 antibodies (CD28.2; BD Biosciences). The purified T cells were stimulated at a concentration of 1 × 10^6^ cells/ml RPMI 1640/5% human AB serum/pen-strep for 24 h, and TCRαβ and CD20 expression as well as cell death were analyzed by flow cytometry. Annexin-V (BioLegend) and live/dead staining (Thermo Fisher Scientific, MA, USA) were used to measure early and late apoptosis, respectively. The purified T cells were also stimulated for 4 days to induce cell proliferation and CD20 expression measured on proliferated (CFSE-low and CFSE-intermediate) and non-proliferated (CFSE-hi) T cells by flow cytometry as described above.

### Antigen stimulation of T cells

Cryopreserved CFSE-labeled PBMC from healthy donors were thawed and B cells depleted using a kit from Stem Cell Technologies, according to manufacturer. Complete removal of B cells was ensured by flow cytometry prior to cultivation of 500,000 PBMC in 20 µg/ml myelin oligodendrocyte glycoprotein (MOG; AnaSpec Inc, CA, USA), 2.5 × 10^6^ candida albicans cells/ml (CA; InvivoGen, CA, USA), 1.5 µg/ml varicella-zoster virus antigen from infected cell extracts (VZV; Medix Biochemica Group, MO, USA), or RPMI 1640/5% human AB serum/pen-strep with no additives for 6 days. Hereafter, PBMC were stained for flow cytometry and CD20 expression measured on proliferated (CFSE-low) and non-proliferated (CFSE-hi) T cells. The experiment was performed three independent times with a total of five individuals.

### Statistical analysis

To compare *MS4A1*-mRNA levels between CD20^−^ and CD20^+^ T cells, a student’s t-test was applied. For statistical analysis of CD20^+^ T cell frequency and CD20 MFI following FoxO1-inhibition, a Friedman test and *post hoc* Dunn’s test for multiple comparisons was performed. In the Dunn’s test the mean rank of unstimulated cells was compared to the mean rank of each of the concentrations applied. To compare CD20^+^ T-cell frequencies in the proliferation studies, paired t-tests were performed.

### Ethics

All participants gave informed, written consent to participation. The study was approved by the regional scientific ethics committee (protocol number H-17005703 and H-16047666).

## Results

### T cell trogocytosis of CD20

To confirm the ability of human CD20^−^ T cells to receive CD20 from B cells and hence develop into CD20^+^ T cells through trogocytosis, we cultured isolated CD20^−^ T cells with autologous antigen-primed B cells (**Figures 1A–D**). Within 1 h, we observed that CD20 (**Figure 1E**) together with a selected set of B-cell surface molecules, including CD21, CD22, and CD180 (**Figure 1F**), were transferred to the surface of the T cells. CD21, CD22, and CD180 were only detected on T cells that acquired CD20 (**Figure 1F**); not on the remaining CD20^−^ T-cell population (**Figure 1G**).

If all CD20^+^ T cells in the blood of humans are a product of trogocytosis, a co-transfer of other B-cell molecules would be expected (34, 35). Analyzing the co-expression of CD20 with other B-cell markers on T cells from healthy donors showed that only CD20 was expressed on human blood T cells, not the B-cell markers CD21, CD22, and CD180 observed in the trogocytosis assay (**Figures 1H–J**). As the scenario may differ in tissue during inflammation, we additionally measured the expression of CD20 and other B-cell markers on T cells in the CSF of newly diagnosed untreated patients with relapsing-remitting multiple sclerosis (RRMS). As observed in the blood, neither CD21, CD22 nor CD180 was co-expressed with CD20 on CD20^+^ T cells in CSF (**Figures 1K–M**). The flow cytometry experiments were performed three independent times; representative examples are shown.

### True naïve CD20^+^ T cells

**Figure 2:**
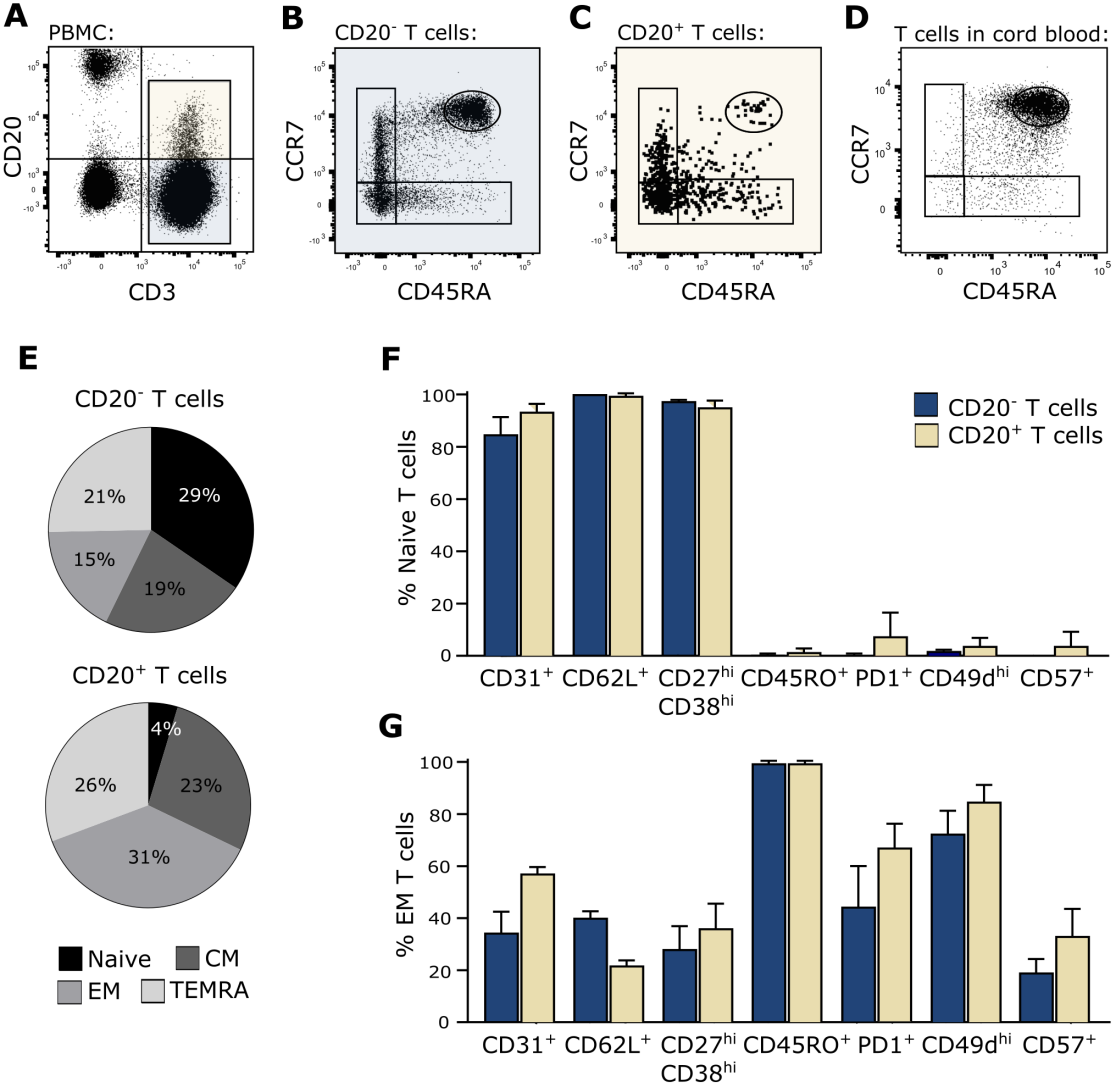
True naïve CD20^+^ T cells. **(A–D)** Flow cytometry dot plot example of naïve T-cell identification in blood. The gating strategy include gating of lymphocytes, single cells, live cells, and CD20^−^ and CD20^+^ T cells as shown in the CD3/CD20-plot in **(A)**. Naïve CD20^−^ T cells **(B)** and CD20^+^ T cells **(C)** in peripheral blood and naïve T cells in cord blood **(D)** were defined as CD45RA^++^CCR7^++^. **(E)** Distribution of naïve (CD45RA^++^CCR7^++^), central memory (CM; CD45RA^−^CCR7^+^), effector memory (EM; CD45RA^−^CCR7^−^), and terminally differentiated T cells (TEMRA; CD45RA^+^CCR7^−^) in the CD20^−^ and CD20^+^ T population in the blood. **(F, G)** Staining of various surface markers associated with a naïve or memory phenotype on **(F)** CD45RA^++^CCR7^++^ naïve T cells and **(G)** CD45RA^−^CCR7^−^ effector memory T cells from three healthy donors. Mean + SD is shown.

In the process of trogocytosis, T cells interact with B cells and become activated by signals induced through the TCR. Therefore, T cells that have gone through trogocytosis are antigen-experienced. To further investigate the possibility that CD20^+^ T cells arise from trogocytosis, we measured the activation and differentiation status of CD20^+^ T cells in the blood of healthy individuals. For this, PBMCs were stained with fluorescence labeled antibodies identifying CD20^+^ and CD20^−^ T cells along with the differential markers CD45RA and CCR7 and analyzed the samples by flow cytometry. To ensure proper identification of naïve T cells according to CD45RA and CCR7, we included staining of cord blood cells, which represents a naïve phenotype. This gating strategy identified a population of blood CD45RA^++^CCR7^++^ CD20^+^ T cells, corresponding to the naïve T cell population found in cord blood (**Figures 2A–E**). To confirm that these blood T cells were truly naïve, they were concomitantly stained with a range of antibodies against surface markers frequently used to identify antigen-naïve T cells (CD31, CD62L, CD27/CD38) and antigen-primed T cells (CD45RO, PD-1, CD49d, CD57). This showed that naïve T cells identified both within the population of CD20^+^ and CD20^−^ T cells stained positive for CD31, CD62L, and CD27^hi^CD38^hi^ and negative for CD45RO, PD-1, CD49d, and CD57, strengthening the classification as a true naïve CD20^+^ T-cell population (**Figure 2F**). As a staining control of activation and differentiation markers, effector memory T cells (CD45RA^−^CCR7^−^) are shown (**Figure 2G**). The data shown are from three independent experiments with *n* = 3 healthy donors.

### Endogenous CD20 production

**Figure 3:**
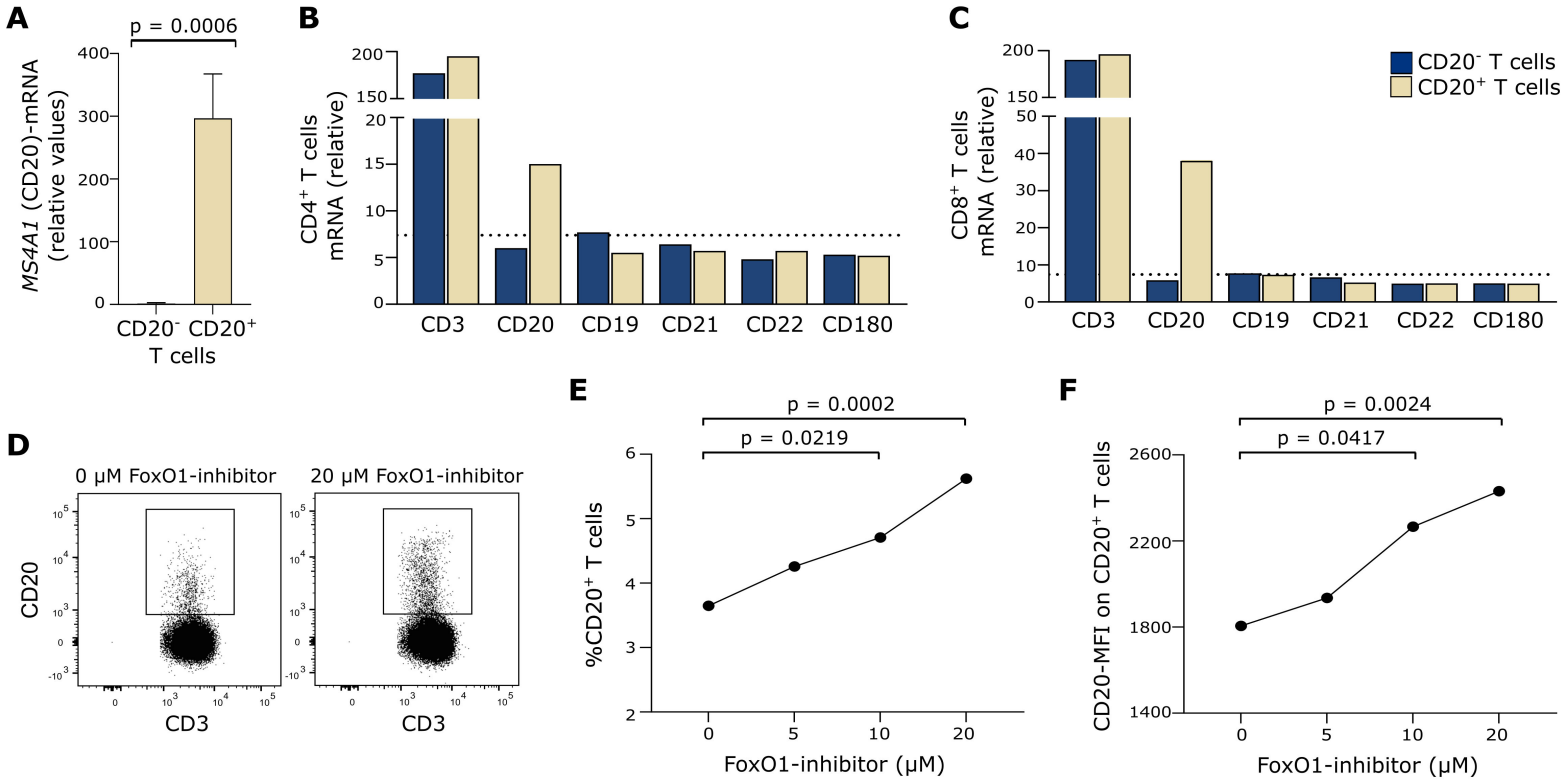
Endogenous CD20 production. **(A)** qPCR measurement of *MS4A1* in CD20^−^ and CD20^+^ T cells from three healthy donors. Mean+SD is shown. **(B, C)** Affymetrix analysis of B-cell–associated surface markers in fluorescence-activated cell sorted CD20^−^ and CD20^+^ T cells; both in **(B)** CD4^+^ and **(C)** CD8^+^ T cells. *MS4A1* is denoted as CD20. Dotted line shows the signal median of all Affymetrix-measured transcripts. **(D)** Flow cytometry dot plot example of CD20 expression on T cells cultured in 0 µM or 20 µM FoxO1-inhibitor. **(E)** Frequency of T cells expressing CD20 after 48 h of culture with 0–20 µM FoxO1-inhibitor (AS1842856) from six healthy donors. **(F)** Mean fluorescence intensity (MFI) of CD20 on CD20^+^ T cells after 48 h of culture with 0–20 µM FoxO1-inhibitor from six healthy donors.

As our data indicate that CD20 on T cells may not only originate from trogocytosis in humans, we next measured endogenous *MS4A1*-mRNA, which translates into CD20 protein. For this, CD20^+^ and CD20^−^ T cells were isolated by fluorescence-activated cell sorting and qPCR applied. In contrast to CD20^−^ T cells, *MS4A1*-mRNA was detected in CD20^+^ T cells (**Figure 3A**). Also, an Affymetrix analysis of sorted cells showed that *MS4A1*-mRNA was measurable in both CD4^+^ and CD8^+^ CD20^+^ T cells in contrast to CD19, CD21, CD22, and CD180 (**Figures 3B, C**), strengthening our qPCR data.

CD20 expression is regulated both by epigenetic and transcription factors (28). The half-time of CD20 protein in human primary resting B cells is 71 h; hence, transcriptional activity of *MS4A1* is to be expected within this timeframe (36). FoxO1 is a negative *MS4A1* transcription factor, and the FoxO1-inhibitor AS1842856 has been shown to induce CD20 expression in B cells (37). To investigate if CD20^+^ T cells have endogenous CD20 production, we therefore incubated T cells with AS1842856 for 48 h and measured the frequency of CD20^+^ T cells and the CD20 surface expression level (MFI). This showed that FoxO1 inhibition induced both the frequency of CD20^+^ T cells and the MFI of CD20 (**Figures 3D–F**), further indicating endogenous CD20 production. The experiment was performed three independent times with a total of six healthy individuals.

### CD20 expression on proliferating T cells

**Figure 4:**
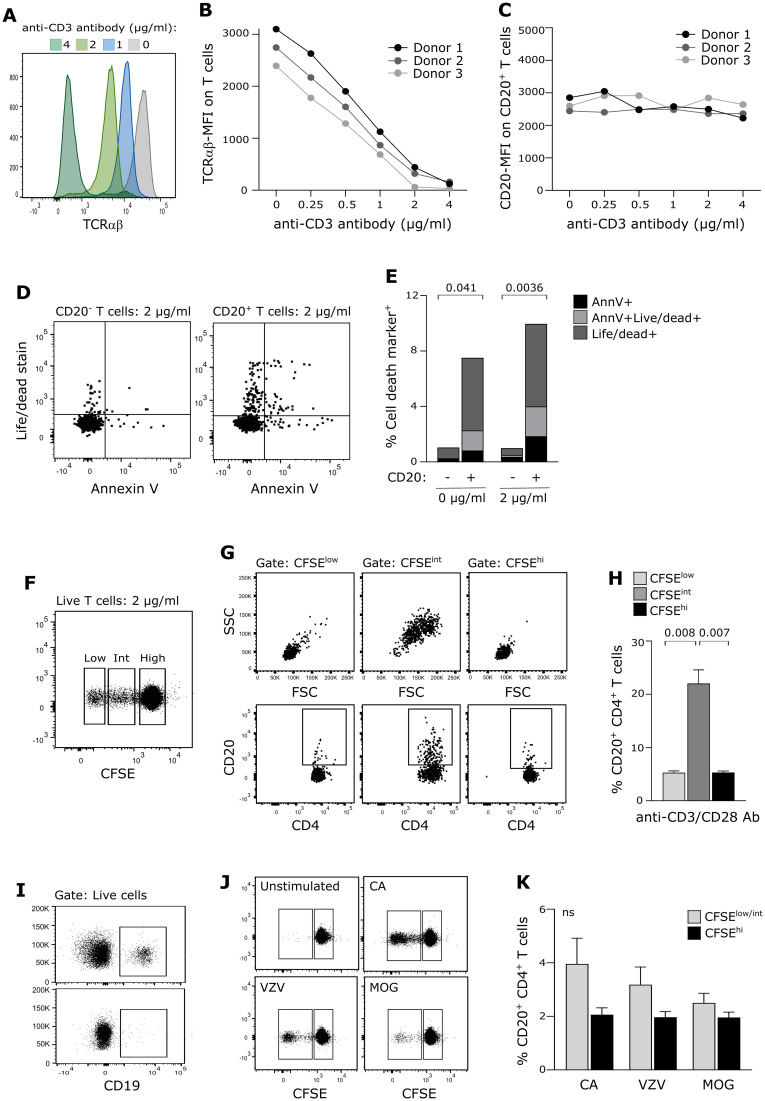
CD20 expression on proliferating T cells. **(A–C)** Surface expression level (MFI) of **(A, B)** TCRαβ and **(C)** CD20 on T cells stimulated with a range of anti-CD3 antibodies and 1 µg/ml anti-CD28 antibodies for 24 h of three healthy donors. **(D, E)** Evaluation of cell death in CD20^−^ and CD20^+^ T cells following 24 h of antibody-stimulation. Positive annexin V–staining indicates early apoptosis, the live/dead-staining marker implies late apoptosis. Mean value of three donors is shown. **(F–H)** Proliferation of T cells from three healthy donors following 4 days of antibody stimulation. **(F)** Flow cytometry dot plot example showing T-cell proliferation (CFSE dilution). **(G)** Flow cytometry dot plot example of cell blasting (upper panel) and CD20 expression (lower panel) of CD4^+^ T cells in the CFSE-high, CFSE-intermediate, and CFSE-low T-cell population. **(H)** Frequency of CD20^+^CD4^+^ T cells following antibody-induced proliferation. Mean + SD is shown. **(I–K)** Proliferation of T cells from five healthy donors following 6 days of antigen-stimulation. **(I)** PBMC before (upper panel) and after (lower panel) B-cell depletion. **(J)** Flow cytometry dot plot example showing T-cell proliferation (CFSE dilution) in response to candida albicans (CA), varicella-zoster virus antigen (VZV) and myelin oligodendrocyte glycoprotein (MOG). **(K)** Frequency of CD20^+^CD4^+^ T cells in response to antigen-induced proliferation. Mean + SD is shown.

To further analyze endogenous production of CD20 in CD20^+^ T cells, we investigated whether CD20 on CD20^+^ T cells is diluted following antigen-induced cell proliferation. For this analysis to be conclusive, a stable surface expression of CD20 is a prerequisite, that is, CD20 must not be shed or internalized following TCR priming. Likewise, CD20^+^ T cells must not suffer from excessive activation-induced cell death. To ensure this, we stimulated purified T cells from three healthy donors for 24 h with increasing concentrations of plate-bound anti-CD3 and anti-CD28 antibodies and observed that CD20 was stably expressed on the surface of T cells in contrast to TCRαβ, which was strongly downregulated (**Figures 4A–C**). Staining of the T cells with a life/dead stain and annexin-V as a marker of early apoptosis showed that CD20^+^ T cells were more prone to activation-induced cell death than their CD20^−^ counterparts (**Figures 4D, E**). A previous study has shown a transient increase in CD20 expression on isolated T cells after 4 days of *in-vitro* stimulation in non-human primates (9). We therefore stimulated purified and CFSE-labeled T cells for 4 days with anti-CD3 and anti-CD28 antibodies and observed an increased CD20-expression in the early proliferation state of T cells where the cells increase in size (blasts) (**Figures 4F–H**; CFSE-intermediate population). Following cell retraction, we observed a normalized CD20-expression pattern of the T cells (**Figures 4F–H**; CFSE-low population).

To validate that CD20 is not diluted on the surface of proliferated T cells in a more physiologically appropriate setting, we next purified and CFSE-labeled PBMCs from five healthy donors and removed all B cells from the samples (**Figure 4I**). Candida albicans (CA), varicella-zoster virus antigen (VZV), or myelin oligodendrocyte glycoprotein (MOG) was added, and the cells were cultured for 6 days (**Figure 4J**). Due to low antigen-reactivity for some donors, we analyzed only non-proliferated and proliferated T cells (i.e., CFSE-intermediate T cells were included in the proliferated population). This experiment confirmed that CD20 is not diluted on daughter cells (**Figure 4K**).

## Discussion

Trogocytosis is a fundamental process in eukaryotic organisms and is involved in cell communication in the immune system (29). Following TCR:MHC-interaction between a T cell and a B cell, MHC can be transferred to the recipient T cell along with B-cell membrane fractions, including other B-cell surface molecules (30, 34, 35). Donor-derived proteins likely retain their biological function when transferred to the surface of the recipient cell; the recipient cell hereby gains novel functions, and in certain cases the donor cell loses function or even dies (30, 32). Studies of both humans and rodents have indicated that T cells expressing CD20 possess a particular proinflammatory phenotype (1, 17); however, whether CD20 expressed on T cells is acquired through trogocytosis or is an intrinsic property of the T cell is highly debated. A recent study showed that murine CD20^+^ T cells developed as a result of trogocytosis, in which TCR: MHCII-interaction between T and B cells led to the transfer of B-cell–expressed CD20 to the T cell. Knocking out B cells (µMT mice) led to deprivation of CD20^+^ T cells only to be restored upon adoptive transfer of CD20^+^ B cells (17). Along this line, mouse CD20^+^ T cells did not express the CD20 transcript *MS4A1* (17). Trogocytosis is therefore likely the driving mechanism of CD20^+^ T cell formation in mice.

Studies appearing in the literature are beginning to question whether CD20 expressed on T cells exclusively originates from trogocytosis in humans, as is observed in mice. To investigate this, we previously analyzed whether humans were born with CD20^+^ T cells, which would suggest that CD20^+^ T cells were an inherited cell lineage. As other research groups have also observed, we found that CD20^+^ T cells were absent in the cord blood of newborn individuals (1, 5). This observation either contradicts CD20^+^ T cells being a cell lineage and hence supports their origin through trogocytosis or implies that the CD20^+^ T cell lineage develops later in life. To explore the possibility of trogocytosis as the origin of human CD20^+^ T cells in our current study, we primed human B cells with antigen and cultured them with autologous human CD20^−^ T cells. Within 1 h, the T cells acquired B cell-derived CD20 along with a series of other B-cell markers, namely, CD21, CD22, and CD180, consistent with the process of trogocytosis. To establish that human CD20^+^ T cells solely are a product of trogocytosis, we next measured co-expression of CD20 on T cells with the B-cell markers CD21, CD22, and CD180 in the blood of healthy donors and CSF of patients shortly after experiencing their first attack in multiple sclerosis as a representative of an inflamed environment. Surprisingly, we did not detect any of the B-cell associated surface markers on CD20^+^ T cells in blood or CSF. A prerequisite of trogocytosis is the interaction of TCR on the T cell with MHC on the B cell, inducing T-cell activation and MHC-transfer to the recipient T cell. In a previous study, we found that only a minor fraction of CD20^+^ T cells express HLA-DR (MHCII), which is in agreement with a range of other human studies (2, 3, 5). Together with the observation that CD21, CD22, and CD180 are not co-expressed with CD20 on T cells, this may imply that other mechanisms in addition to trogocytosis are involved in the development of CD20^+^ T cells in humans.

As CD20^+^ T cells developed through trogocytosis represent a primed T-cell subset, we measured the differentiation status of circulating CD20^+^ T cells in healthy individuals. Although the majority of CD20^+^ T cells analyzed were previously primed, we identified a population of apparently true naïve T cells. This observation contradicts that all human CD20^+^ T cells originate from trogocytosis and supports the hypothesis that additional pathways contribute to the development of CD20^+^ T cells.

To explore the possibility that human CD20^+^ T cells can also arise through endogenous production of CD20, we measured expression of the CD20-transcript *MS4A1* by qPCR in resting CD20^+^ and CD20^−^ T cells. This showed *MS4A1* only in CD20^+^ T cells, a finding verified by various other human studies (2, 3). To confirm endogenous expression of *MS4A1* in resting CD20^+^ T cells, we performed a gene chip analysis and found expression of *MS4A1* but not the transcripts of the B-cell markers CD21, CD22, and CD180 in CD20^+^ T cells. These mRNA data support our protein findings of circulating human CD20^+^ T cells. *MS4A1*-transcription is regulated by various transcription factors including, FoxO1, which is a negative regulator of *MS4A1* expression (28, 37). Inhibition of FoxO1 led to an increase in CD20 on T cells, further supporting the observation of endogenous CD20-production in CD20^+^ T cells.

If CD20 is endogenously produced, CD20 expression on T cells would be inherited to daughter cells. An *in-vitro* study of stimulated T cells from non-human primates showed a transient increase in CD20 expression following 4 days of stimulation, an increase that was reduced after prolonged cell culture (9). To investigate if the same applies to human T cells, we stimulated isolated T cells for 4 days with anti-CD3 and CD28 antibodies and observed a similar increase in the blasting phase of primed T cells, only to decrease again to the level of non-proliferating T cells when blasted cells retracted again. TCR-induced blasting of T cells is part of early proliferation and drives a major increase in the total RNA content and protein synthesis of the cells (38). Our result indicates that CD20 is likely among the produced proteins in the blasting phase, ensuring proper CD20 expression on daughter cells. To strengthen this finding, we performed a similar stimulation assay where B cell-depleted PBMC cultures were incubated with different antigens and CD20 expression measured on antigen-stimulated T cells. In coherence with our observations in the anti-CD3/CD28 antibody stimulated cultures, this showed that surface-expressed CD20 was retained on the surface, not diluted, following T-cell proliferation. We did not prolong the cultivation of cells in any of the two assays as was done in the non-human primate study, since CD20^+^ T cells are more prone to activation-induced cell death, introducing a bias. Altogether, these experiments show that CD20^+^ T cells do not lose CD20 expression following proliferation and that the CD20 surface expression level depends on how and how long cells are stimulated in culture.

In support of our finding that CD20 is endogenously produced by human T cells is the observation that CD20^+^ T-cell lymphomas are a malignancy arisen from neoplastic transformation of a normal CD20^+^ T cell and that these cells expand clonally (39, 40).

In conclusion, our data suggest that human CD20^+^ T cells can develop through trogocytosis, but in contrast to rodent species, they also have the ability to endogenously produce CD20 and transfer expression to daughter cells. One could speculate that trogocytosis of CD20 is a way for T cells to quickly gain the functional properties that CD20 confers during immune responses, for example, to improve their proinflammatory reactivity toward antigens. Clinically, our findings possibly support the use of a combination therapy in which CD20 expression on T-cell lymphomas can be increased to enhance the treatment efficacy of anti-CD20 antibody therapies.

## Data Availability

The data that support the findings of this study can be shared by request from any qualified investigator. Sharing requires approval of a data transfer agreement in accordance with GDPR and Danish data protection regulation.
