# Supplementary material for: The origin of human CD20+ T cells: a stolen identity?
Source: Front Immunol. 2024 Nov 22;15:1487530. doi: 10.3389/fimmu.2024.1487530 (PMC11621209; doi:10.3389/fimmu.2024.1487530)
Supplement: Supplementary file 1 [file Table1.docx]

**Supplementary table 1**

| Antibody target | Conjugate | Clone | Company |
| --- | --- | --- | --- |
| Trogocytosis assay | | | |
| CD3 | BV421 | UCHT1 | BioLegend |
| CD19 | BV605 | HIB19 | BioLegend |
| CD20 | PE-Cy7 | 2H7 | BioLegend |
| CD21 | APC-Fire750 | Bu32 | BioLegend |
| CD22 | PE | S-HCL-1 | BioLegend |
| CD180 | APC | RP105 | BioLegend |
| Naïve T cell analysis | | | |
| CD3 | BV605 | UCHT1 | BioLegend |
| CD20 | PE-Cy7 | 2H7 | BioLegend |
| CD25 | BV421 | M-A251 | BioLegend |
| CD27 | BV421 | O323 | BioLegend |
| CD28 | BV605 | CD28.2 | BioLegend |
| CD31 | BV605 | WM59 | BioLegend |
| CD45RA | FITC | HI100 | BioLegend |
| CD45RO | BV421 | UCHL-1 | BioLegend |
| CD49d | BV421 | 9F10 | BioLegend |
| CD57 | BV421 | HNK-1 | BioLegend |
| CD62L | BV421 | DREG-56 | BioLegend |
| CD127 | BV605 | A019D5 | BioLegend |
| CD197 (CCR7) | PE | G043H7 | BioLegend |
| CD279 (PD-1) | BV605 | EH12.2H7 | BioLegend |
| FoxO1 assay | | | |
| CD3 | FITC | UCHT1 | BioLegend |
| CD20 | PE-Cy7 | 2H7 | BioLegend |
| CD19 depletion control | | | |
| CD3 | BUV395 | UCHT1 | BD |
| CD19 | BV605 | HIB19 | BioLegend |
| CD20 | PE-Cy7 | 2H7 | BioLegend |
| CD20 stability and cell death assay | | | |
| TCRαβ | APC | IP26 | BioLegend |
| CD4 | BV421 | RPA-T4 | BioLegend |
| CD8 | BV605 | RPA-T8 | BioLegend |
| CD20 | PE-Cy7 | 2H7 | BioLegend |
| Annexin-V | PE |  | BioLegend |
| Proliferation assay: anti-CD3/CD28 antibody stimulation | | | |
| TCRαβ | APC | IP26 | BioLegend |
| CD4 | BV421 | RPA-T4 | BioLegend |
| CD8 | BV605 | RPA-T8 | BioLegend |
| CD20 | PE-Cy7 | 2H7 | BioLegend |
| Proliferation assay: antigen stimulation | | | |
| CD3 | BUV395 | UCHT1 | BD |
| CD4 | BV421 | RPA-T4 | BioLegend |
| CD8 | APC | RPA-T8 | BioLegend |
| CD20 | PE-Cy7 | 2H7 | BioLegend |
